# Design and 3D FEM Analysis of a Flexible Piezoelectric Micromechanical Ultrasonic Transducer Based on Sc-Doped AlN Film

**DOI:** 10.3390/s22218100

**Published:** 2022-10-22

**Authors:** Qinghua Ren, Junhong Chen, Xin Liu, Songsong Zhang, Yuandong Gu

**Affiliations:** School of Microelectronics, Shanghai University, Shanghai 200444, China

**Keywords:** flexible, PMUT, Sc-doped AlN, 3D-FEM, simulation

## Abstract

In this paper, a flexible piezoelectric micromachined ultrasonic transducer (PMUT) based on Scandium (Sc)-doped Aluminum Nitride (AlN) film was designed and modeled by the three-dimensional finite element method (3D-FEM). The resonant frequency of 218.1 kHz was reported. It was noticeable that a high effective electromechanical coupling coefficient (k^2^_eff_) of 1.45% was obtained when a combination of a flexible PI and a thin Si layer was used as the PMUT supporting structure layer. Compared with a pure Si supporting layer counterpart, the coupling coefficient had been improved by 110.68%. Additionally, the increase of Sc doping concentration in AlN film further enhanced the device electromechanical coupling coefficient and resulted in an improvement for transmitting/receiving sensitivity of the proposed flexible PMUT. When the doping concentration of Sc reached 30%, the emission sensitivity was as large as 1.721 μm/V, which was 2.86 times greater than that of conventional AlN film-based PMUT. The receiving sensitivity was found to be 2.11 V/KPa, which was as high as 1.23 times the performance of an undoped device. Furthermore, the bending simulation result showed that the proposed flexible PMUT device can maintain a good mechanical stability when the bending radius is greater than 1.5 mm. The simulation of sound field characteristics demonstrated that the flexible PMUT based on AlScN could receive stable sound pressure signals under the bending radius of 1.5 cm.

## 1. Introduction

Micromachined ultrasonic transducers (MUTs) have been widely studied for many acoustic applications including diagnostic ultrasound [1], ultrasonic imaging [2], fingerprint identification [3] and so on, taking advantages of miniaturization, lower power consumption, and manufacturability [1]. MUTs have become a promising alternative to traditional ultrasonic transducers. There are two categories of MUT: capacitive MUTs (CMUTs) [4] and piezoelectric MUTs (PMUTs) [5]. Compared with CMUTs, PMUTs based on flexural vibrations can achieve satisfactory sensitivity without ultrahigh bias voltage and tiny gaps [6]. Traditional PMUTs are either fabricated on a rigid structure or made of conventional bulk piezoelectric materials. Haoran Wang et al. designed a 4 μm thick ceramic lead zirconate titanate (PZT) device with a significantly higher piezoelectric coefficient and lower stress than those of devices based on sol–-gel or sputtered PZT [7]. Dan Gong et al. designed an AlN-based piezoelectric micromachined ultrasonic transducer as a wireless power receiver [8]. Bernard Herrera et al. reported on the first demonstration of acoustic power transfer using Aluminum Nitride (AlN) Piezoelectric Micromachined Ultrasonic Transducer (PMUT) arrays [9]. The transmission distances of the AlN-PMUT arrays are suitable for intra-body powering applications. Although these PMUTs had the advantages of low cost, high density, and easy integration with CMOS circuits, their rigid structure limited the application of devices on irregular planes [10].

Compared with a rigid PMUT, a flexible PMUT has the advantages of weight, size, adaptability, and portability. Therefore, many studies on flexible PMUTs have been reported in recent years. In 2013, Yi Yang et al. studied a novel flexible piezoelectric ultrasound transducer based on MEMS technology, which was used in medical imaging with conformal contact with skin surfaces [11]. In 2015, Zhe Wang et al. proposed a novel flexible piezoelectric micromachined ultrasound transducer based on PZT and a polyimide substrate [12]. Although the above two PMUTs have a high electromechanical coupling coefficient and flexibility, strict packaging is required. In 2019, Sina Sadeghpour et al. fabricated a flexible PMUT multi-array structure based on silicon-on-insulator (SOI) technology [13]. This multi-array structure can be wrapped in a cube to achieve omnidirectional measurement, but its manufacturing process is complicated. In 2020, Wei Liu et al. proposed a flexible piezoelectric micro ultrasonic transducer (PMUT) based on a laser-processed substrate. However, the transmitted and received signal intensity of the PMUT device was weak [14]. In addition to PZT [15] and polyvinylidene fluoride (PVDF) [16,17] mentioned above, there are many piezoelectric materials for flexible PMUTs, such as zinc oxide (ZnO) and aluminum nitrous (AlN). PZT has the largest piezoelectric coefficient (d_33_ = 60 pm/V) and a large dielectric constant. However, the toxic lead content in PZT may cause health and environmental issues [18]. ZnO has a high piezoelectric coefficient (d_33_ = 9.93 pm/V) [19], but its piezoelectric properties are unstable under extreme conditions. Although PVDF is a flexible material, it has a low piezoelectric coefficient and is incompatible with CMOS processes. Interestingly, AlN has a moderate piezoelectric coefficient (d_33_ = 5.1 pm/V) [20] and satisfactory insulating and dielectric properties [21]. Furthermore, Sc-doping can improve the piezoelectric coefficient of AlN. The performance improvement depends largely on the level of the doping concentration of Sc. To the best of our knowledge, only limited studies on AlN-based flexible PMUTs have been reported by far, and a systematic optimization of such a flexible structure still remains unreported. For example, Sheng Sun et al. reported a flexible AlN PMUT device with the advantages of high flexibility, miniaturization, and easy integration [22]. However, a further discussion on the stability of the device and the associated bending state were missing.

In this paper, a PMUT based on Sc-doped AlN (AlScN) film was proposed and modeled by 3D FEM in COMSOL software. Firstly, the characteristic frequency, electromechanical coupling coefficient, and static sensitivity of PMUT on three substrates were compared. The FEM results indicated that the optimized performance at the resonant frequency of 218.1 kHz was obtained with the composite supporting structures of a PI and a thin Si layer. Secondly, the enhancement of Sc-doping in AlN on the piezoelectric device performance was systematically analyzed. With an increase in Sc concentration, the static transmitting (Tx) and receiving (Rx) sensitivity of PMUT were proportionally improved with only a minimum amount of frequency drift. The optimal Sc-doping concentration of 30% was explored by conducting the bending simulation of the proposed flexible PMUT. The resultant PMUT structure demonstrated a good flexibility for flexible applications. Finally, the sound field characteristics of flexible AlScN PMUTs were studied in air domains. Stable sound pressure signals at a bend radius of 1.5 cm validated the good Tx/Rx function of our proposed flexible ScAlN PMUT.

## 2. Materials and Methods

### 2.1. Device Structure

**Figure 1:**
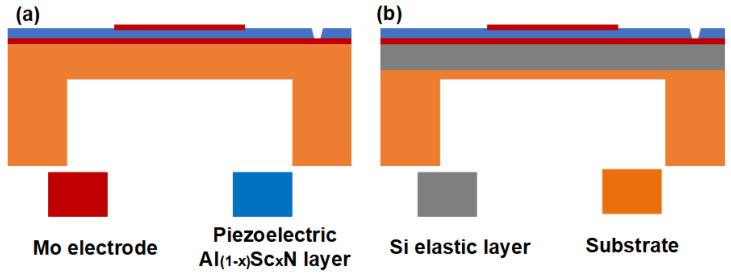
(**a**) The cross section of a single traditional flexible PMUT; (**b**) the cross section of the single flexible PMUT proposed in this paper.

The conventional PMUT structure mainly consists of a substrate, a bottom electrode, a piezoelectric layer, and a top electrode. The schematic cross section of an individual PMUT is shown in Figure 1a. When Si is selected as the substrate, PMUT is rigid, while when PI is selected as the substrate, PMUT is flexible. However, compared with the rigid PMUT, the frequency characteristics and piezoelectric performance of the flexible PMUTs are significantly attenuated. For example, Wei Liu et al. prepared a flexible PMUT based on ZnO [23]. The simulation results of this literature showed that the resonance frequency of PMUT was 2.32 MHz, and the electromechanical coupling coefficient was about 0.815%. The frequency of a rigid PMUT with Si substrate at the same size is 8.12 MHz, and the electromechanical coupling coefficient is 1.22%. It can be seen that changing the substrate from rigid Si to flexible PI can cause a decrease in frequency and electromechanical coupling coefficient of the PMUT.

Therefore, a special flexible PMUT structure was proposed in this paper. The PMUT consists of a piezoelectric sandwich structure with a flexible PI substrate containing a thin silicon support layer. The schematic cross section of an individual PMUT in this paper is shown in Figure 1b. A 1 μm thick Al_1-x_Sc_x_N film is used as the piezoelectric layer. The Al_1-x_Sc_x_N film is sandwiched between two 0.2 μm molybdenum (Mo) electrodes. The substrate used here is a 3.5 μm thick Si supporting layer on a PI membrane with a thickness of 1 μm. A cavity of 100 μm in depth is set below the PI membrane substrate. This novel flexible substrate containing a thin silicon support layer is used not only to achieve its flexibility and bendability, but also to ensure the stability of the PMUT’s characteristic frequency and piezoelectric properties. Structural parameters of the PMUT unit are shown in Table 1. The PMUT frequency designed in this paper is suitable for air applications around 200 kHz.

### 2.2. 3D-FEM Model and Mesh-Dependency Analysis

**Figure 2:**
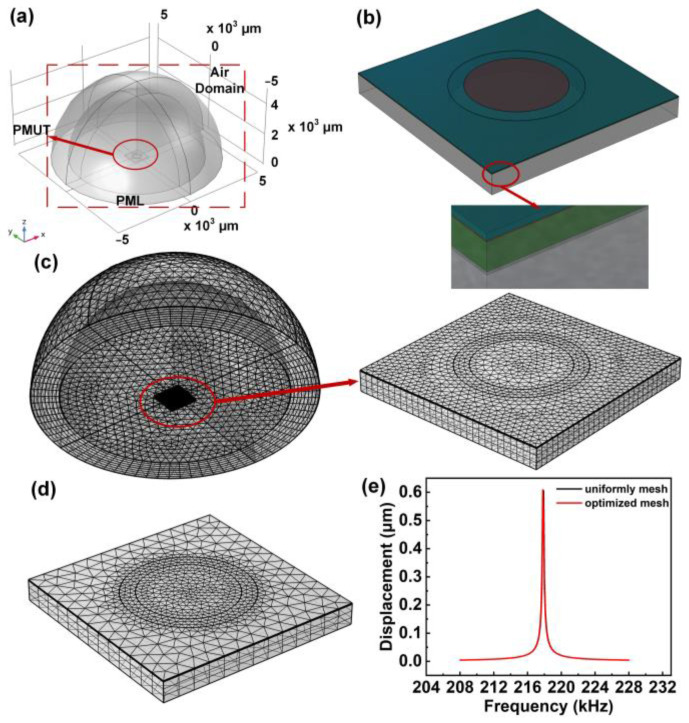
(**a**) The FE geometry of PMUT under air, (**b**) The details of each layer of PMUT element; (**c**) A mesh partition of FE geometry and zoomed-in view of PMUT element; (**d**) The results after mesh optimization of PMUT element; (**e**) The frequency–displacement curves of the uniformly segmented and optimized mesh.

3D-FEM for simulation of complex structures is the most efficient and intuitive method for modeling and analyzing the resonance mode shapes and electromechanical-acoustic performance of PMUT and its arrays [24]. The 3D-FEM model of a single PMUT is simulated in COMSOL Multiphysics 5.6. The models consider the sandwiched structure, the cavity under the passive layer, the substrate, the passivation layer, and the air domain. Figure 2a illustrates the FE geometry of PMUT with the abovementioned geometric parameters in an air domain. The PMUT element, for solid mechanical simulation in which the AlN layer and Mo electrodes are highlighted in blue and red, respectively, as shown in Figure 2b. In addition, the details of the other layers are shown in the inset, where green represents Si and gray represents PI. Material models were obtained from the COMSOL material library. The material properties used are shown in Table 2.

The applied elements of the physical field are set as follows: PMUT belongs to the solid mechanics module. Piezoelectric material belongs to the electrostatics module. The air domain belongs to the pressure acoustics module. The perfect matching layer (PML) is added to reduce the influence of reflected acoustic waves on the results. The thickness of PML is set as 1 mm, which is larger than the half wavelength of PMUT. In the simulation, the case that the bottom of a single PMUT is fixed and the sound wave propagates upward without reflection is simulated. Therefore, the boundary conditions are set as follows: The bottom of the PMUT is set as the roll support. The bottom of the hemispherical air domain is the boundary of the hard sound field. Both the cavity and the PML are set as the free boundary. Moreover, a 1 V excitation voltage was applied to the top electrode while grounded in the bottom electrode.

The mesh of PMUT is divided into two steps: first, the x-y plane is divided into free triangles, and then the z-axis direction is swept. The size of the mesh is set as ultra-fine, and the distribution of swept is set as 3 layers. When the model of the air domain is meshed, free triangular meshes are used for the inner air domain and swept meshes are used for the PML to reduce computation time. To ensure the accuracy of simulation results, the overall mesh size of the air domain should be less than one-fifth of the wavelength. A mesh partition and zoomed-in view are shown in Figure 2c. However, too small a mesh size will lead to a large amount of computation. Therefore, mesh optimization is indispensable. The most critical part of the model is the thin film above the cavity, which requires a super-fine mesh. The mesh size can be increased appropriately for the rest of the model. The results after mesh optimization are shown in Figure 2d. Figure 2e compares the frequency–displacement curves of the uniformly segmented and optimized mesh. It can be seen that the frequency and displacement amplitude of the two segmentation methods are basically consistent. The model in the subsequent paper will use the mesh generation method in this section.

### 2.3. Mode Shape Selection

**Figure 3:**
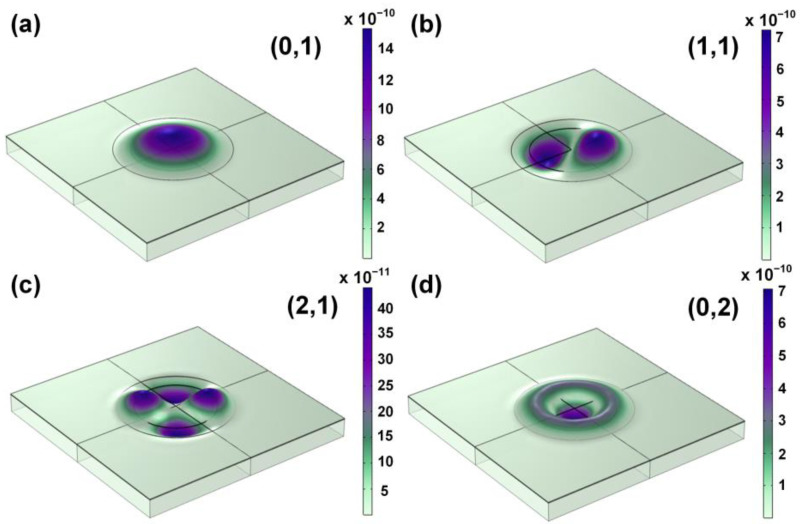
First four typical vibration modes of the proposed PMUT. (**a**) (0,1) mode; (**b**) (1,1) mode; (**c**) (2,1) mode; (**d**) (0,2) mode.

Frequency is one of the key parameters of PMUT, so the frequency of device vibration mode is the first to be obtained. The vibration modes of PMUT were obtained by eigenfrequency analysis. Figure 3 shows the first four typical vibrational modes of the proposed PMUT device. The purple color represents maximum displacements, and the white color represents minimum displacements. The displacements of (1,1) and (2,1) modes offset each other, and the modes are not easy to excite in the medium. Furthermore, the higher-order modes are not quickly excited in the medium. Both the (0,1) and (0,2) modes have no displacement offset, but the (0,2) mode is challenging to excite in the medium [25]. Therefore, the most suitable mode of our PMUT is the first-order vibration mode (0,1), as shown in Figure 3.

### 2.4. Evaluation of PMUT Properties

The electromechanical coupling coefficient (k^2^_eff_) can characterize the energy conversion efficiency of PMUT. Without considering the PMUT geometry and excitation mode, the value of the k^2^_eff_ can be obtained through the following steps: Firstly, the admittance diagram of PMUT is obtained from frequency domain simulation. Then, the resonant frequency (f_r_) and anti-resonant frequency (f_a_) of the (0,1) mode are extracted from the admittance diagram. Finally, it can be obtained through the following calculation formula [26]:

(1) $$k_{\mathrm{eff}}^{2}=1-{\left(\frac{f_{r}}{f_{a}}\right)}^{2}$$

Moreover, the static transmitting sensitivity (d_r_) and static receiving sensitivity(s_r_) of PMUT are obtained through frequency simulation [27]. The d_r_ is defined as the film center displacement under unit voltage excitation. In the sweep range, the film can produce the maximum displacement at the resonance frequency, which mainly utilizes the positive piezoelectric effect of PMUT. In contrast to d_r_, s_r_ converts the received sound pressure into voltage using the inverse piezoelectric effect. The s_r_ is defined as the voltage amplitude generated by PMUT per 1 kPa pressure. In the simulation, the voltage of 1 V is added to the top and bottom electrodes as static excitation and the pressure of 100 kPa as boundary load.

For flexible PMUT devices, mechanical stability under bending conditions is crucial. Because flexible devices are mainly used on uneven surfaces, the unstable structure of PMUT will lead to significant errors in measurement results. The bending performance of PMUT is tested by steady-state analysis. In bending deformation, PMUT will produce different degrees of strain. When the deformation exceeds the critical value, the film will rupture. The strain of each layer structure can be calculated by the formula below [14]:

(2) $$\varepsilon_{\mathrm{material}}=\frac{t_{\mathrm{material}}}{2r+t_{\mathrm{material}}}$$

where t_material_ is the material thickness and r is the bending radii. The curved film will not crack under the condition of ε ≤ δ_max_, where δ_max_ is the elongation of the material [27].

The sound field characteristics of PMUT are studied by using time-domain analysis. This analysis mainly reflects the dynamic transmission and reception characteristics of PMUT in the air. The simulation follows: apply a specific pulse signal to the top electrode to make the film emitting PMUT produce bending vibration. The sound pressure generated by the vibration of the transmitter is transmitted through the air to the receiver PMUT. The receiver PMUT is excited by sound pressure to vibrate, thus generating an electrical signal [28]. The dynamic sensitivity performance of PMUT is expressed by the relative pulse receiving sensitivity level M. Its mathematical expression is as follows: [29]

(3) $$M=20\mathrm{lg}\left(\frac{U_{\max}}{U_{0}}\right)$$

where U_max_ is the maximum value of receiving voltage and U_0_ is the value of transmitting voltage.

## 3. Results and Discussions

### 3.1. Basic Characteristics of PMUT

**Figure 4:**
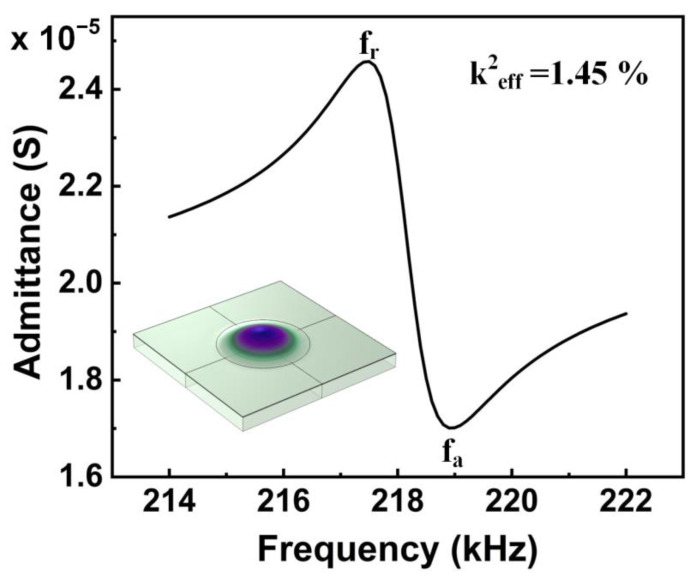
The admittance of the proposed flexible PMUT.

Different substrate materials will affect the mechanical and acoustic properties of PMUT [30]. This paper studies the frequency domain simulation of PMUT on three different substrates. The substrates selected here are Si, PI with a thin layer of Si, and PI. Figure 4 shows the admittance of PMUT based on the PI with a thin Si substrate. PMUT based on a pure PI substrate has a frequency of only 70 kHz. Therefore, the cavity radius of PMUT with PI substrate is reduced to 180 μm to obtain a comparable frequency. The piezoelectric performance results were obtained from frequency-domain simulation. The calculation analysis is shown in Table 3. It can be seen from the table that the k^2^_eff_ of the PMUT based on the PI with a thin layer of Si substrate can reach 1.45%, which is 136% higher than the PMUT of the PI substrate. At the same time, the sensitivity of static receiving and transmitting is obviously better than that of PMUT on PI substrate. Notably, compared with the flexible PMUT based on Si substrate, the k^2^_eff_ of the flexible PMUT based on thin Si substrate is increased by 110%. The main reason for the decreasing frequency is that Young’s modulus and Poisson’s ratio of PI are smaller than that of Si. To sum up, the proposed PMUT shows excellent piezoelectric performance and is expected to replace the flexible PMUT with a pure PI substrate.

**Figure 5:**
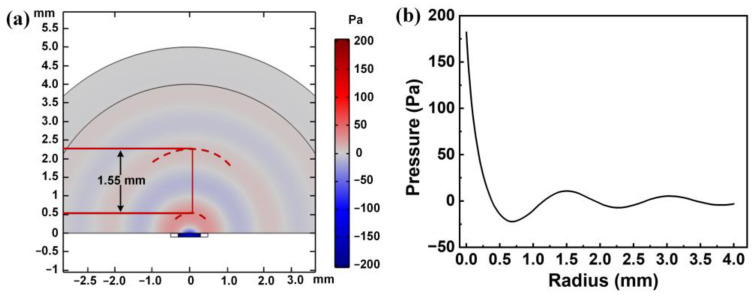
(**a**) Cross section of the spherical air domain 3D results; (**b**) The acoustic wave sizes at different distances on the central axis of PMUT.

Furthermore, to observe the acoustic characteristics of PMUT, a 4 mm spherical air domain is added to the original PMUT model. Sound waves generated by PMUT can be simulated in the air domain by coupling the sound field with the structural physical field. Figure 5a shows the cross section of the 3D results, from which a rough wavelength (λ) of a sound wave of 1.55 mm is estimated. Theoretically, the wavelength can be calculated using the formula λ = c/f, where c is the sound speed in the air medium, which is 340 m/s. The f is frequency, which is 218.1 kHz. Therefore, λ is calculated to be 1.56 mm, which is consistent with the simulation result. The acoustic wave sizes at different distances on the central axis of PMUT are further calculated, as shown in Figure 5b. As the radius increases, the attenuation of sound waves increases, and the location of the peaks and troughs can be easily predicted from the acoustic curve.

### 3.2. Piezoelectric Characteristics of PMUT with Different Sc Doping Concentrations in Al_1-x_Sc_x_N Piezoelectric Materials

It is well known that Sc doping in AlN can significantly improve the piezoelectric properties of AlN. From the atomic point of view, the size of the Sc atom is larger than the Al atom. Lattice distortion occurs when a substitution occurs. Furthermore, Sc is less electronegative than Al, which partly explains the larger piezoelectric effect of Al_1-x_Sc_x_N [31]. The change in crystal structure will affect the elastic constant and piezoelectric coefficient of the material. The following formula can calculate the elastic constants and piezoelectric coefficients [32].

(4) $$C_{11}\left(x\right)=374.1\left(1-0.882x+0.602x^{2}\right)\mathrm{GPa}$$

(5) $$C_{12}\left(x\right)=128.6\left(1+0.400x-0.082x^{2}\right)\mathrm{GPa}$$

(6) $$C_{13}\left(x\right)=100.3\left(1+0.793x-0.481x^{2}\right)\mathrm{GPa}$$

(7) $$C_{33}\left(x\right)=351.7\left(1-1.160x-0.256x^{2}\right)\mathrm{GPa}$$

(8) $$C_{44}\left(x\right)=111.6\left(1-0.848x+1.369x^{2}\right)\mathrm{GPa}$$

(9) $$e_{15}\left(x\right)=-0.313\left(1-0.296x-1.687x^{2}\right)C/m^{2}$$

(10) $$e_{31}\left(x\right)=-0.593\left(1+0.311x+0.971x^{2}\right)C/m^{2}$$

(11) $$e_{33}\left(x\right)=1.471\left(1+0.699x+4.504x^{2}\right)C/m^{2}$$

where C_mn_ is the elastic constant, e_mn_ is the piezoelectric coefficient, mn represents the direction, and x is the Sc doping concentration.

**Figure 6:**
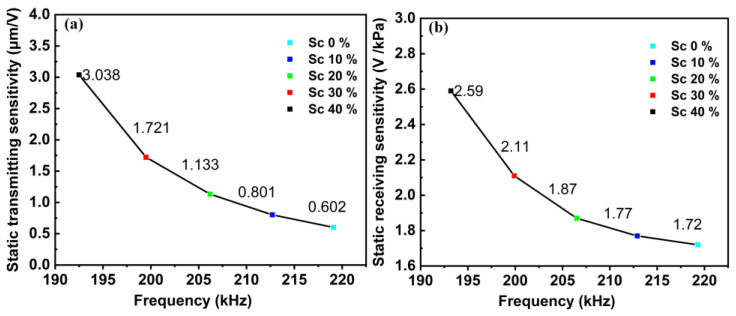
(**a**) Static transmitting sensitivity of the proposed PMUT with different Sc doping concentrations in Al_1-x_Sc_x_N piezoelectric material; (**b**) Receiving sensitivity of the proposed PMUT with different Sc doping concentrations in Al_1-x_Sc_x_N piezoelectric material.

The piezoelectric properties of PMUT with different Sc doping were obtained by changing the piezoelectric materials in the PMUT model. The results of the d_r_ are shown in Figure 6a. When the Sc doping content is 0%, the maximum d_r_ is only 0.602 μm/V, while when the Sc doping content is 30%, the maximum d_r_ is 1.721 μm/V. The sensitivity is increased by 2.86 times, which is consistent with the theoretical results.

However, the reception sensitivity of PMUT is also affected by the dielectric constant. The increase of constant dielectric results in a decrease in receiving sensitivity. Its calculation formula is [33]:

(12) $$\varepsilon_{33,f}=\left(9.0\pm 0.6\right)+\left(20.9\pm 0.6\right)x+\left(47.5\pm 20.1\right)x^{2}$$

(13) $$\varepsilon_{33}=\varepsilon_{33,f}-\frac{e_{33}^{2}}{\varepsilon_{0}C_{33}}$$

where x is the doping concentration, ε_33,f_ is the relative dielectric coefficient (clamped), ε_0_ is the vacuum permittivity, e_33_ is the piezoelectric coefficient, and C_33_ is the elastic stiffness at the constant electric field. According to Formulas (12) and (13), the permittivity of different Sc doping concentrations in Al_1-x_Sc_x_N can be obtained. The results show that the doping of Sc increases the dielectric constant of PMUT. In theory, this should lead to a lower s_r_. Interestingly, the simulation results show that increasing Sc doping concentration can significantly increase the s_r_ of PMUT. The increase of s_r_ is since the piezoelectric coefficient caused by Sc doping is greater than the dielectric constant, which increases the receiving sensitivity [34]. The receiving sensitivity at different Sc doping concentrations is shown in Figure 6b. When the doping concentration is 0, the receiving sensitivity is 1.72 V/kPa, and when the Sc doping concentration is 30%, the emission sensitivity is 2.11 V/kPa. The s_r_ increased by 1.23 times.

Although increased Sc concentration significantly improves the emission and receiving sensitivity of PMUT, the material density of Al_1-x_Sc_x_N decreases due to the lattice distortion caused by the incorporation of Sc atoms. The decrease in density of Al_1-x_Sc_x_N will reduce the characteristic frequency of the PMUT. In this paper, a large piezoelectric response is obtained by considering the frequency and piezoelectric response, and the frequency reduction is less than 10%. Finally, Al_0.7_Sc_0.3_N was selected as the preferred piezoelectric material with d_r_ of 1.72 μm/V, s_r_ of 2.11 V/kPa, and frequency of 200 kHz.

### 3.3. Mechanical Properties

**Figure 7:**
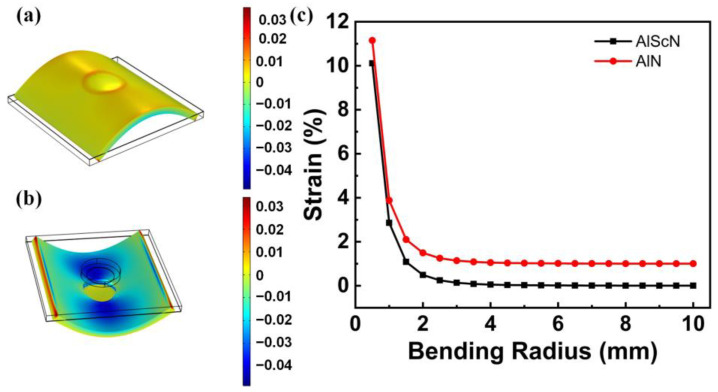
(**a**) The stress distribution on the upper surface of PMUT under bending conditions; (**b**) The stress distribution on the lower surface of PMUT under bending conditions; (**c**) The maximum strain curves under different bending radii with Al_0.7_Sc_0.3_N and AlN as piezoelectric materials.

In order to characterize the flexibility of the proposed PMUT, the strain distribution of the PMUT under different bending radii was simulated by using the 3D-FEM method. In order to simulate the bending state, the boundary conditions of the PMUT elements need to be changed. The boundary conditions change from the bottom roller support to the fixed displacement of the left and right sides. The fixed displacements on the left and right sides are set opposite magnitude along the x-axis. The bending radius of PMUT can be calculated by displacement. Figure 7a,b shows the strain distributions on the upper and lower surfaces of PMUT in bending states. It can be seen that the upper surface is mainly affected by tensile strain, and the lower surface is mainly affected by compressive strain. Furthermore, the maximum strain is centralized around the cavity of the PMUT. The maximum strain distribution with increasing the bending radius based on the Al_0.7_Sc_0.3_N and AlN piezoelectric materials was demonstrated in Figure 7c. It can be found that PMUT based on Al_0.7_Sc_0.3_N piezoelectric material has a lower strain change at the same bending radius than that of PMUT based on AlN piezoelectric material. It means that the flexibility of PMUT is improved with Al_0.3_Sc_0.7_N being used as a piezoelectric layer. In addition, when the bending radius decrease from 10, 8, 6, to 4 mm, the corresponding maximum strain values are 0.0033%, 0.006%, 0.0139%, and 0.0528%, respectively, as clearly shown by the black line in Figure 6c. It is well known that Si is the most easily fractured brittle material. The PMUT proposed in this paper contains a thin Si layer of PI. Therefore, the maximum material elongation of the whole PMUT is determined by Si and the value reported is 0.25% [35]. The PMUT device will break down once the strain exceeds 0.25%. As a result, our PMUT can keep better mechanical stability as long as the bending radius is larger than 2.5 mm.

### 3.4. Dynamic Analysis

**Figure 8:**
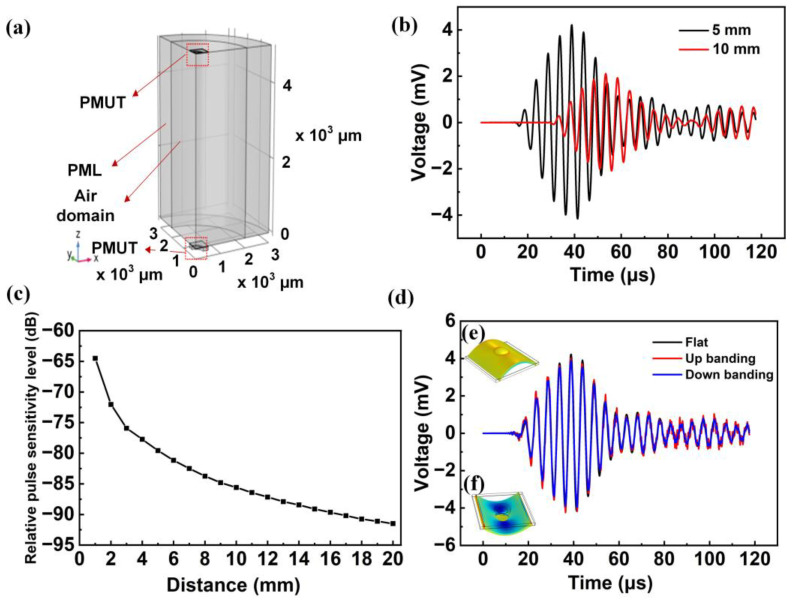
(**a**) A quarter model diagram of proposed PMUT with a receiving PMUT device, air domain, and perfect matching layer respectively; (**b**) The voltage waveform received by the receiver with the reflector at distances of 5 mm and 10 mm under transient simulation; (**c**) The relative pulse sensitivity level shift at different distances of the reflector; (**d**) Comparison of receiving voltages of PMUT under different bending states.

The dynamic transmission and reception characteristics of the proposed flexible PMUT based on Al_0.7_Sc_0.3_N piezoelectric materials in the air are studied by time-domain analysis. A quarter of the structure is modeled here to reduce a large amount of computational cost. Figure 8a demonstrates the representation model. In the model, the inner part of the air domain is divided by free tetrahedral mesh, and the outer perfectly matched layer (PML) is divided by sweeping. The PMUT located at the bottom is a transmitter, and the PMUT located at the top is a receiver. The transmitter is excited by a 5-cycle-sinusoidal pulse with an amplitude of 40 V_pp_ and a frequency of 200 kHz.

Figure 8b shows the voltage signal of the receiving PMUT at distances of 5 mm and 10 mm, respectively. The dynamic sensitivity performance of PMUT is expressed by the relative pulse receiving sensitivity level M. The result is shown in Figure 8c. Clearly, the M decreases with increasing the distance between the transmitting PMUT and the receiving PMUT. When the distance increased to 20 mm, the relative pulse sensitivity level decreased to −91.5 dB. This result is better than that of PVDF-based flexible PMUT [10] at the same distance.

**Figure 9:**
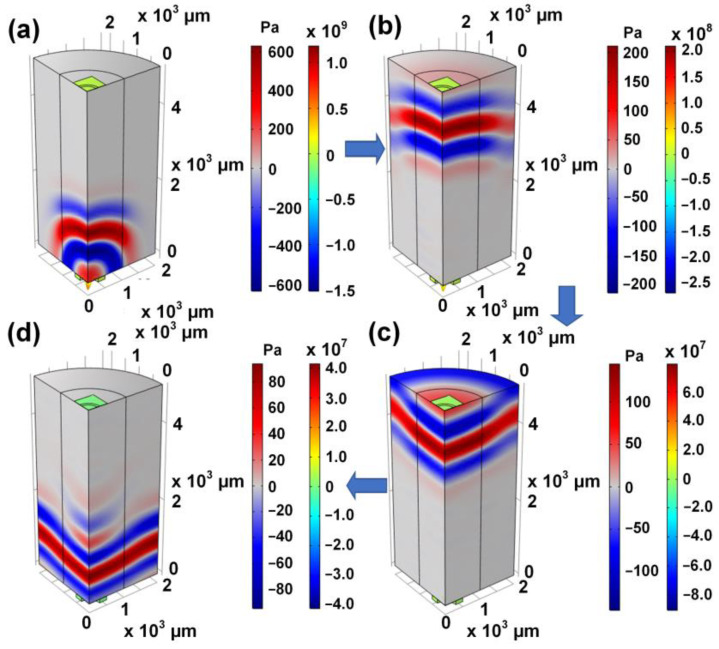
The process by which sound waves travel through the air.

Finally, the reception performance of the proposed PMUT at different bending states is studied. The bending states tested here are upbending state, downbending state, and the flat state. The bending radius was set to 1.5 cm. The test results are demonstrated in Figure 8d. The result indicates that the flexible PMUT can maintain stable piezoelectric performance in a bending state. The process by which sound waves travel through the air is shown in Figure 9. Transmitters generate sound waves, which travel through the air to receivers. In the process, the sound pressure in the air changes. In addition, part of the sound wave is reflected back to the original transmitter and then received. Obviously, the proposed PMUT emits uniform sound waves in the air with a wavelength of about 1.5 mm, which is consistent with the resonant frequency.

## 4. Conclusions

This paper proposed a flexible PMUT with Sc-doped AlN film as a piezoelectric material. The performance of the PMUT was systematically investigated using 3D-FEM. Simulation results showed that the best performance was obtained in PMUT with the flexible PI substrate added with a thin layer of Si. In addition, the simulation result showed that the piezoelectric properties of PMUT would be significantly improved by increasing the Sc doping concentration. Compared with the piezoelectric film without Sc doping, the static transmission and static receiving sensitivity were increased by 2.86 times and 1.23 times, respectively, when the Sc concentration was set to 30%. The mechanical tests showed that the flexible PMUT can maintain mechanical stability when the bending radius exceeds 1.5 mm. These results demonstrated that the proposed flexible PMUT based on Sc-doped AlN with PI substrate added to a thin Si layer can achieve high k^2^_eff_, high sensitivity, and high flexibility. Furthermore, the acoustic field characteristics of PMUT were also studied. The simulation results showed that the relative pulse sensitivity level of PMUT based on Al_0.7_Sc_0.3_N was −91.5 dB at the distance of 20 mm, which is 18 dB higher than that of PMUT based on PVDF. The received signals in different bending states showed that the proposed flexible PMUT based on Al_0.7_Sc_0.3_N can receive stable signals in convex, concave, and flat states. In summary, the proposed flexible PMUT has potential applications in flexible ultrasonic range finders, position sensing, and other fields.

## Figures and Tables

**Table 1:** Structural parameters of the proposed PMUT.

Parameter	Value
The thickness of top Mo	0.2 μm
The thickness of AlN/Al_1-x_Sc_x_N	1 μm
The thickness of bottom Mo	0.2 μm
Thickness of Si	3.5 μm
The depth of the cavity	100 μm
Radius of cavity	300 μm
Radius of Si	500 μm
The radius of bottom Mo	500 μm
The radius of top Mo	230 μm

**Table 2:** Material properties of AlN, Si, Mo, and PI in 3D-FEM models.

Property	Symbol	AlN	Si	Mo	PI
Density (kg/m^3^)	𝜌	3300	2329	10,200	1300
Poisson ratio	𝜐	0.3	0.064	0.3	0.37
Young’s modulus (GPa)	𝛶	330	170	312	3.1
Elastic stiffness constants c^E^_ij_ (×10^11^ N m^−1^)	c_11_ c_12_ c_13_ c_33_ c_44_ c_66_	4.1 1.49 0.99 3.89 1.25 1.305	-	-	-
Piezoelectric constants e_ij_ (C m^−2^)	e_31_ e_33_ e_15_	−0.58 1.55 −0.48	-	-	-
Dielectric permittivity	ε/ε_0_	10	-	-	-

**Table 3:** Properties of PMUT with different substrates.

Substrate Types	Si	PI with a Thin Layer of Si	PI
Resonant frequency f (kHz)	226.5	218.1	203.9
Effective electromechanical coupling k^2^_eff_ (%)	1.31	1.45	1.07
Static transmitting sensitivity $d_{s}$ (μm/V)	0.547	0.602	0.247
Static receiving sensitivity $s_{r}$ (V/kPa)	1.70	1.72	0.6

## Data Availability

The data presented in this study are available on request from the corresponding author.
